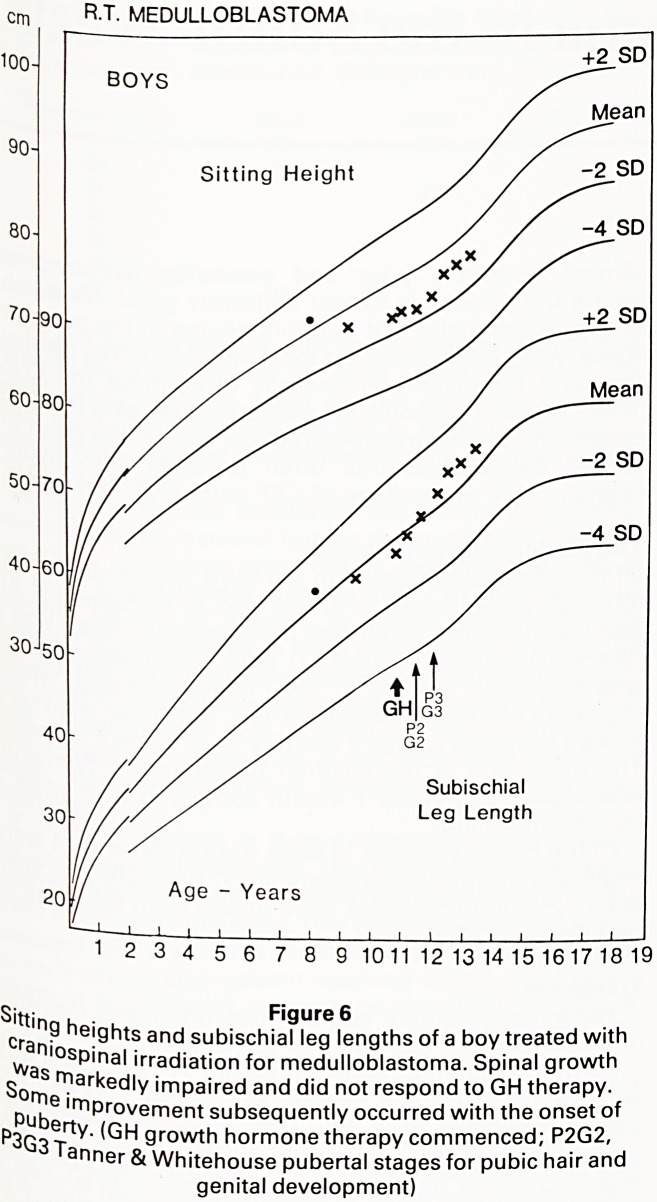# Growth and Growth Hormone Secretion Following Cranial and Craniospinal Irradiation in Children with Malignant Disease

**Published:** 1988

**Authors:** P. S. Ward

**Affiliations:** Bristol Children's Hospital


### Growth and growth hormone secretion in children treated with cranial and craniospinal irradiation for cancer and leukaemia

**P. S. Ward**
Bristol Children's Hospital

### INTRODUCTION

As the prognosis for many childhood malignancies has improved the late effects of chemotherapy and radiotherapy have become more important. One of the principal differences between adults and children is that children grow. The Bristol paediatric oncology and paediatric endocrinology services have collaborated to investigate the effects of chemotherapy and craniospinal irradiation on the longitudinal growth of survivors.

Figure 1 shows the longitudinal height-attained growth chart of a child who grew poorly following treatment for a medulloblastoma. Our experience, and that of others, is that children treated for brain tumours, head and neck tumours and some leukaemic children are at risk of subsequent growth impairment (1,2,3). Some of these children can be shown in conventional pharmacological stimulation tests to become growth hormone (GH) deficient (1,4). Others grow poorly despite having apparently normal GH reserves. The object of our research was to investigate this phenomenon further.

**Figure 1**
Longitudinal growth of a girl with medulloblastoma treated with craniospinal irradiation

### RADIOTHERAPY OR CHEMOTHERAPY?

In order to determine whether chemotherapy or radiotherapy is more important in causing subsequent growth failure we investigated the long term growth of 2 groups of children who had been treated for acute lymphoblastic leukaemia. The first group received chemotherapy alone. The second group received chemotherapy and craniospinal irradiation, usually 2400 rad to the head and 1000 rad to the spine. Heights at diagnosis and at approximately annual intervals afterwards were extracted retrospectively from the case notes. Height standard deviation scores (SDS) were calculated for each observation. The results are shown in Figure 2.

**Figure 2**
Height standard deviation scores by years from diagnosis of leukaemia for children treated (a) without and (b) with craniospinal irradiation

Patients who received chemotherapy alone showed no significant change in height SDS in the years following diagnosis and treatment. However, there was a generalised diminution of the height SDS in the years following diagnosis in children who received axial radiotherapy. This suggests that axial irradiation is largely responsible for the growth failure observed in some survivors of childhood malignant disease. There was considerable inter-patient variation in the growth pattern following craniospinal irradiation. All of the patients received the same total radiation dose with similar fractionation and on similar equipment. The reason for the increased susceptibility of some individuals to growth failure remains unclear.

### CRANIOSPINAL IRRADIATION AND BODY PROPORTIONS

The treatment of some childhood malignancies involves the irradiation of the head and spine. Cranial irradiation may result in growth hormone deficiency if a sufficiently large radiation dose is delivered to the hypothalamus. Irradiation of the vertebral column may result in damage to the growth plates and may inhibit normal spinal elongation.

We looked at the standing height, sitting height and subischial leg length of 15 children who had received craniospinal irradiation for a variety of malignant diseases and had subsequently been referred to the paediatric endocrine clinic because of poor growth. Subischial leg length was calculated arithmetically by subtracting the sitting height from the standing height. Height, sitting height and subischial leg length standard deviation scores were calculated for each patient. For each set of observations sitting height SDS was subtracted from subischial leg length SDS to obtain an index of body proportions. In a given population this index is normally distributed around zero. Upper and lower limits have been determined by Tanner and Whitehouse.

Figure 3 shows the results for the 15 children studied. All but one had SDS differences greater than zero. The difference was most marked for children aged 12 years and over. This data makes no allowance for the effect of puberty when the greater part of spinal elongation occurs. However, it is clear that inhibition of spinal growth makes a significant contribution to growth failure following craniospinal irradiation.

**Figure 3**
Subischial leg length SDS less sitting height SDS at the time of referral to the endocrine clinic for children treated with craniospinal irradiation. (P97 97th percentile, P3 3rd percentile)

### SPONTANEOUS AND STIMULATED GROWTH HORMONE SECRETION

Human growth hormone (GH) is secreted episodically especially during slow-wave (stage 4) sleep. Conventionally, GH secretion is assessed by observing the change in serum GH concentration in response to pharmacological stimuli such as insulin-induced hypoglycaemia, intravenous arginine infusion and oral clonidine. Prior to the introduction of biosynthetic GH, the supply of therapeutic human GH was controlled by the Health Services Human Growth Hormone Committee (HSHGHC). GH would not usually be supplied to children who were found to have a peak serum GH concentration greater than 15 mU/L following pharmacological stimulation.

We investigated the hypothesis that following cranial irradiation some children retain the capacity to secrete GH in response to pharmacological stimulation but fail to secrete GH physiologically and therefore grow poorly.

We studied spontaneous GH secretion during EEG-monitored sleep in a group of short children including some who had previously received cranial irradiation. The peak serum GH concentration observed during slow wave sleep was compared with that observed following insulin-induced hypoglycaemia and intravenous arginine infusion.

We found that the peak serum GH concentration observed during sleep correlated significantly with that following hypoglycaemia (r=0.64) and arginine infusion (r=0.57). We identified three slowly growing children who had peak serum GH concentrations greater than 15 mU/L following pharmacological stimulation but failed to achieve this concentration during slow wave sleep. Figure 4 illustrates the results for one of these children who had been treated for a medulloblastoma. All three had received cranial or craniospinal irradiation [5].

**Figure 4**
Spontaneous and pharmacologically stimulated GH secretion in a boy treated for medulloblastoma with craniospinal irradiation. Peak serum GH concentration was less than 15 mU/L during sleep but exceeded 15 mU/L following arginine infusion. (w wake)

### PREDICTION OF GH DEFICIENCY FOLLOWING CRANIAL IRRADIATION

Since growth failure not uncommonly follows cranial irradiation, we wondered whether the pattern of growth during the first few years following the diagnosis of malignant disease was predictive of the subsequent development of GH deficiency. We used heights extracted retrospectively from case notes to calculate height SDSs at diagnosis and at approximately annual intervals thereafter for children who had received axial irradiation and had subsequently been shown to have peak serum GH concentrations greater than (normal) or less than (GH deficient) 15 mu/L. Annual height velocities and height velocity standard deviation scores were calculated. The results for the two groups were compared.

Children who were subsequently shown to have low peak serum GH concentrations had a significantly lower mean height velocity SDS in the first two years following diagnosis and showed a significantly greater fall in mean height SDS in the first year (Figure 5). However there was much inter-patient variation and the results for the two groups overlapped. We were unable to identify any single growth parameter within the first year or two of diagnosis which was predictive of the future development of GH deficiency. (European Society for Paediatric Endocrinology, Heidelberg, 1984).

### CONCLUSIONS

Investigating the growth of children treated for cancer and leukaemia is made difficult by the many variables present, the relatively small numbers of patients available for study in any one centre and the length of time necessary for changes to become discernible. Nevertheless it has become apparent that survivors may subsequently grow poorly and that cranial and craniospinal irradiation are largely responsible for this problem [2,6].

GH deficiency may follow cranial irradiation, the probability depending on the total radiation dose received by the hypothalamus. Some children can be shown to have unequivocal classical GH deficiency. Our studies seem to suggest that a few children retain the ability to secrete GH in response to pharmacological stimulation but secrete GH suboptimally during sleep [5]. When only human GH was available for treatment, the HSHGHC imposed strict criteria which had to be met before therapeutic GH could be supplied. Children with peak serum GH concentrations greater than 15 mu/L during pharmacological stimulation tests were usually denied treatment. Now that biosynthetic GH is readily available, albeit at a price, such criteria no longer apply. We have long suspected that the figure of 15 mU/L is too low to distinguish which children are likely to benefit from exogenous GH therapy. We would suggest that biosynthetic GH therapy be considered in poorly growing children who have received cranial irradiation even if they are shown to have a "normal" GH response to pharmacological stimulation. Similar conclusions have been reached by others [7].

It is clear that spinal irradiation impairs spinal growth and contributes to growth failure. The magnitude of this effect may have been overlooked in the past. Impaired spinal elongation may contribute to the disappointing response to GH therapy sometimes seen in GH deficient children who have received craniospinal irradiation. Children with idiopathic GH deficiency may have height velocities of 12 cms per year or more in the first year of treatment. Such rapid height velocities are rarely seen in children who have received craniospinal irradiation. Figure 6 illustrates the sitting heights and

**Figure 5**
Mean height velocity SDS by years from diagnosis of malignant disease for children subsequently shown to have peak serum GH concentrations (a) less than and (b) greater than 15 mU/L. The drift towards zero in group (a) is an artefact caused by the removal from the analysis of the most severely affected children when they were put on GH therapy subischial leg lengths of a boy who received craniospinal irradiation for medulloblastoma and was subsequently shown to be GH deficient. Following the commencement of human GH therapy there was rapid increase in the subischial leg length but little change in the sitting height. However some spinal elongation was observed with the onset of puberty.

This article has not addressed the effect of chemotherapy and radiotherapy on the gonads. Spinal irradiation may result in gonadal damage in both sexes as a result of scatter of the radiation beam. Any resulting abnormalities of sex steroid secretion will have additional implications for the onset of puberty and the pubertal growth spurt.

Much still remains to be learnt about growth impairment in survivors of childhood malignancies but paediatricians should now be aware that the problem exists and should monitor growth as part of the long term follow up of these patients. Ideally the sitting height should be measured in addition to the standing height. Close cooperation between oncologists and endocrinologists offers the best chance for successful investigation and management. Bristol is fortunate in having such a relationship. Unfortunately, in some hospitals, children's heights may still be religiously plotted on growth charts and yet no action be taken when the observations fall progressively through the centiles.

### ACKNOWLEDGEMENTS

I wish to acknowledge the tremendous help and encouragement provided by Drs D. C. L. Savage, Hilary Morgan, A. Oakhill and M. G. Mott. This work was supported by the Cancer and Leukaemia in Childhood Trust.

**Figure 6**

Sitting heights and subischial leg lengths of a boy treated with craniospinal irradiation for medulloblastoma. Spinal growth was markedly impaired and did not respond to GH therapy. Some improvement subsequently occurred with the onset of puberty. (GH growth hormone therapy commenced; P2G2, P3G3 Tanner & Whitehouse pubertal stages for pubic hair and genital development)

### REFERENCES

1. BROWN, I. H., LEE, T. J., EDEN, O. B. et al. (1983) Growth and endocrine function after treatment for medulloblastoma. *Arch. Dis. Childh.* **58**, 722–727.
2. GRIFFIN, N. K. and WADSWORTH, J. (1980) Effect of treatment of malignant disease on growth in children. *Arch. Dis. Childh.* **55**, 600–603.
3. SHALET, S., BEARDWELL, C. G., AARONS, B. M. et al. (1978) Growth impairment in children treated for brain tumours. *Arch. Dis. Childh.* **53**, 491–494.
4. SHALET, S., BEARDWELL, C. G., MORRIS-JONES, P. H. et al. (1976) Growth hormone deficiency after treatment of acute leukaemia in children. *Arch. Dis. Childh.* **51**, 489–493.
5. WARD, P. S. and SAVAGE, D. C. L. (1985) Growth hormone responses to sleep, insulin hypoglycaemia and arginine infusion. *Hormone Res.* **22**, 7–11.
6. HERBER, S. M., KAY, R., MAY, R. et al. (1985) Growth of long term survivors of childhood malignancy. *Acta. Paediatr. Scand.* **74**, 438–441.
7. ROMSHE, C. A., ZIPF, W. B., MISER, A. et al. (1984) Evaluation of growth hormone release and human growth hormone treatment in children with cranial irradiation associated short stature. *J. Pediatr.* **104**, 177–181.

## Figures and Tables

**Figure 1 f1:**
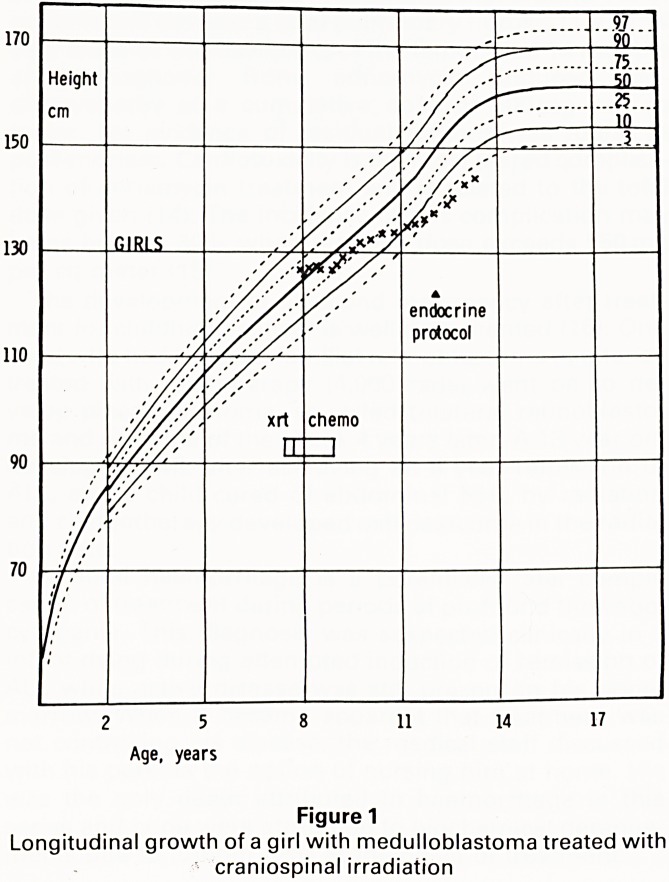


**Figure 2 f2:**
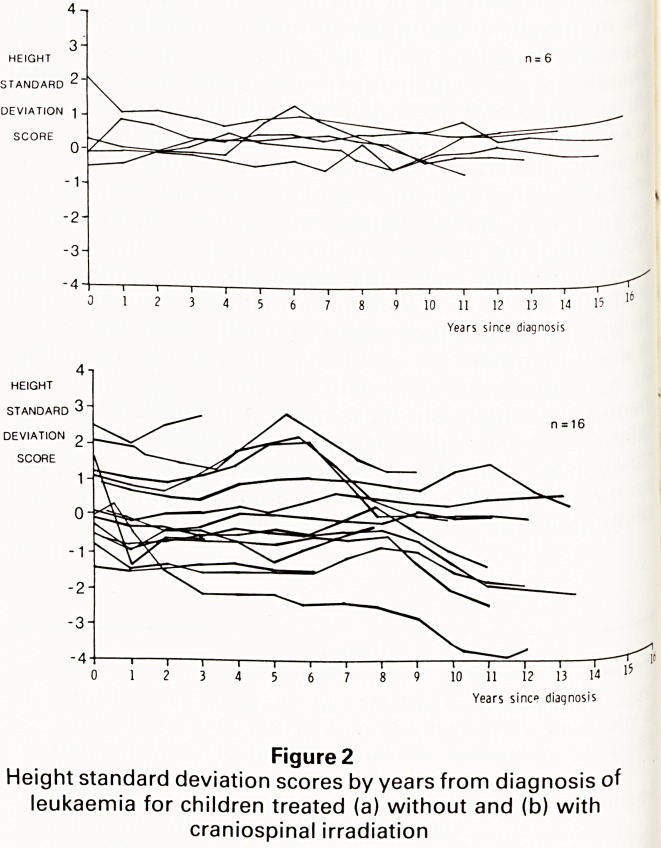


**Figure 3 f3:**
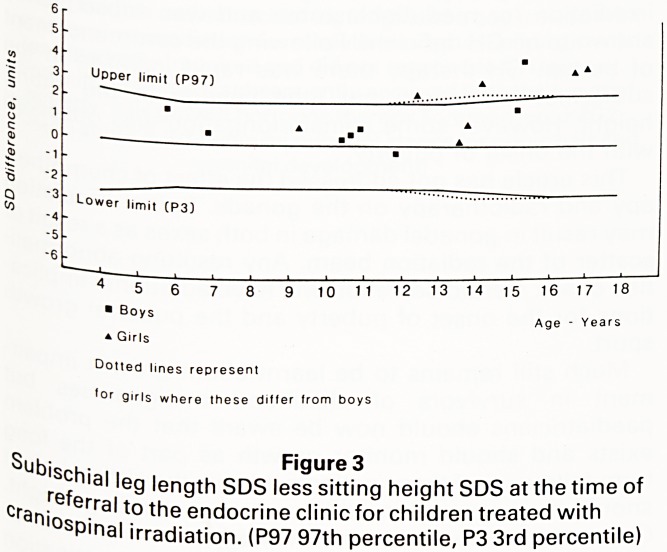


**Figure 4 f4:**
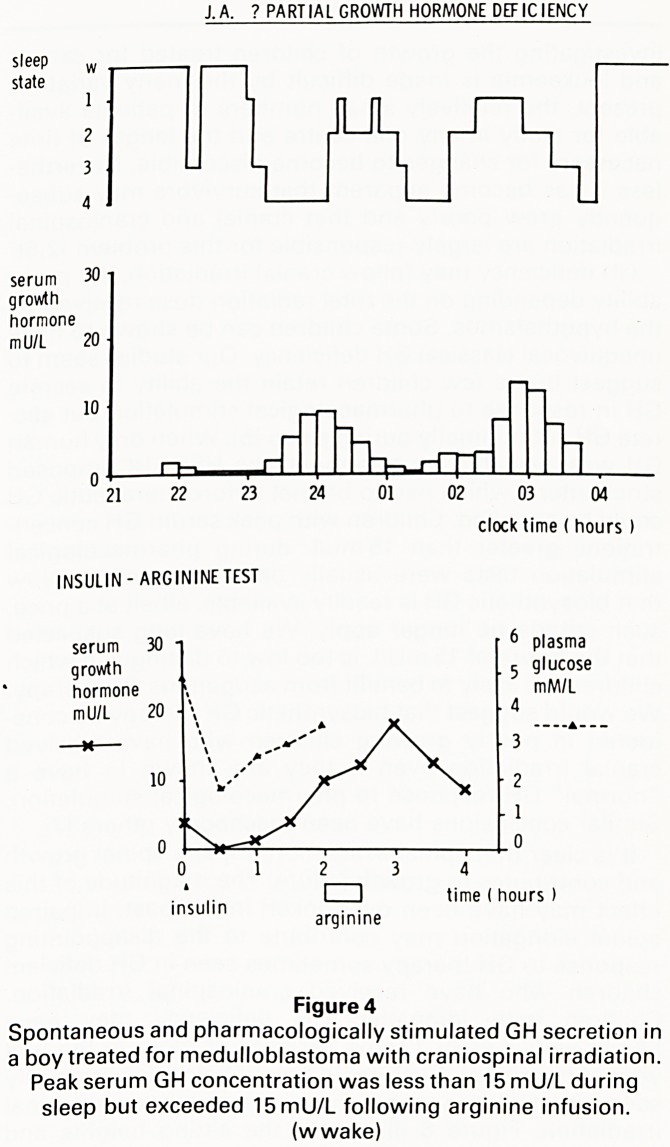


**Figure 5 f5:**
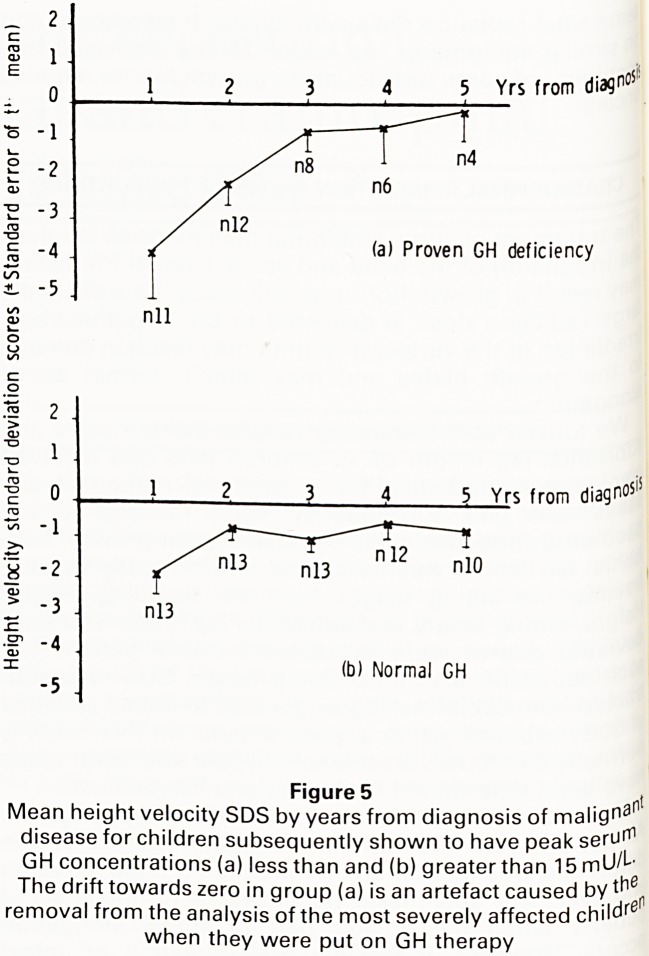


**Figure 6 f6:**